# Molecular evaluation of anti-inflammatory activity of phenolic lipid extracted from cashew nut shell liquid (CNSL)

**DOI:** 10.1186/s12906-018-2247-0

**Published:** 2018-06-11

**Authors:** Marilen Queiroz de Souza, Isabella Márcia Soares Nogueira Teotônio, Fernanda Coutinho de Almeida, Gabriella Simões Heyn, Priscilla Souza Alves, Luiz Antônio Soares Romeiro, Riccardo Pratesi, Yanna Karla de Medeiros Nóbrega, Claudia B. Pratesi

**Affiliations:** 1 Interdisciplinary Laboratory of Biosciences and Celiac Disease Research Center, School of Medicine, University of Brasilia, Asa Norte – CEP 70910900, Brasilia, DF Brazil; 2 Department of Pharmacy, Faculty of Health Sciences, University of Brasilia, Brasilia, DF Brazil; 3 Post-graduate Program in Pharmaceutical Sciences, Faculty of Health Sciences, University of Brasilia, Brasilia, DF Brazil; 4 Post-graduate Program in Health Sciences, Faculty of Health Sciences, University of Brasilia, Brasilia, DF Brazil; 5 Post-graduate Program in Medical Sciences, School of Medicine, University of Brasilia, Brasilia, DF Brazil

**Keywords:** Fenolic lipid, Anacardic acid, Gene expression, Cashew nut shell liquid, CNSL, Anti-inflammatory activity

## Abstract

**Background:**

*Anacardium occidentale L* phenolic lipid (LDT11) is used in traditional medicine as anti-inflammatory, astringent, antidiarrheal, anti-asthmatic and depurative. Phenolic derivatives, such as anacardic acid, extracted from cashew nut shell liquid (CNSL) have demonstrated biological and pharmacological properties, and its profile makes it a candidate for the development of new anti-inflammatory agents.

The objective of the present study was to evaluate the anti-inflammatory profile of a derivative, synthesized from LDT11, on an in vitro cellular model.

**Methods:**

Organic synthesis of the phenolic derivative of CNSL that results in the hemi-synthetic compound LDT11. The cytotoxicity of the planned compound, LDT11, was analyzed in murine macrophages cell line, RAW264.7. The cells were previously treated with LDT11, and then, the inflammation was stimulated with lipopolysaccharide (LPS), in intervals of 6 h and 24 h. The analysis of the gene expression of inflammatory markers (*TNFα, iNOS, COX-2, NF-κB, IL-1β* and *IL-6*), nitric oxide (NO) dosage, and cytokine IL-6 were realized.

**Results:**

The results showed that the phenolic derivative, LDT11, influenced the modulatory gene expression. The relative gene transcripts quantification demonstrated that the LDT11 disclosed an immunoprotective effect against inflammation by decreasing genes expression when compared with cells stimulated with LPS in the control group. The NO and IL-6 dosages confirmed the results found in gene expression.

**Discussion:**

The present study evaluated the immunoprotective effect of LDT11. In addition to a significant reduction in the expression of inflammatory genes, LDT11 also had a faster and superior anti-inflammatory action than the commercial products, and its response was already evident in the test carried out six hours after the treatment of the cells.

**Conclusion:**

This study demonstrated LDT11 is potentially valuable as a rapid immunoprotective anti-inflammatory agent. Treatment with LDT11 decreased the gene expression of inflammatory markers, and the NO, and IL-6 production. When compared to commercial drugs, LDT11 showed a superior anti-inflammatory action.

## Background

**Fig. 1:**
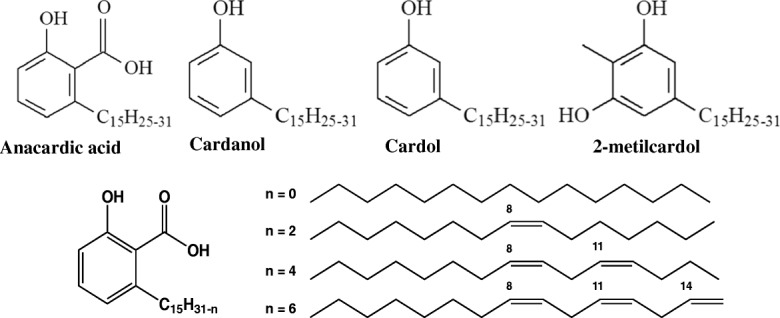
Non-isoprenoid phenolic lipid constituent of the CNSL

Cashew nut shells are considered a residue of the cashew nut processing by the agribusiness. However, for those in search of useful substances from renewable sources it has proven to be valuable bio-based material. The cashew nut shell contains a liquid (CNSL) that is a caustic, viscous oil comprising 25% of the fruit weight *in natura.* CNSL extracted by the cashew processing industry, which separates the almond and the oil, is one of the most abundant sources of non-isoprenoid phenolic lipid, such as anacardic acid, cardol, cardanol and methylcardol (Fig. 1) [1]. The CNSL components, in addition to an aromatic nucleus and several distinct functional groups, has an acyclic side chain containing multiple instabilities in the aliphatic chain, which results in an amphipathic behavior. From a synthetic point of view, CNSL properties characterize it as an extremely versatile material [2].

Nations in South America, Africa, and Asia have for decades use *Anacardium occidentale L.* phenolic lipid, extract from CNSL, in traditional medicine [3–5]. In folk medicine, CNSL is used as anti-inflammatory, astringent, antidiarrheal, anti-asthmatic, depurative and tonic medication. It is also used as diabetes medication [4, 6, 7] and wounds and wart treatment [8–10].

Past research has confirmed that phenolic and semi-synthetic derivatives of CNSL have biological properties [11], such as antibacterial, anti-inflammatory [12–14], and antioxidant activity [15]. Additionally, pharmacological properties included enzymatic inhibition [16, 17] and antiproliferative activity [16, 18]. A recent review by Hemshekhar et al. [19] reinforced anacardic acid multi-target pharmacological profile and its potentiality for the development of new anti-inflammatory drugs.

Inflammation is part of the complex biological response by body tissue to harmful stimuli, caused by infections, injuries or trauma. It is a complicated process regulated by several pro-inflammatory mediators, such as TNF-α, COX-2, iNOS, NF-kB, IL-1β, and IL-6 [20]. The rapid release of pro-inflammatory cytokines by activated macrophages plays a crucial role in triggering local immune response [21]. However, excessive production of inflammatory mediators may be more damaging than the event that triggered the immune response and may be associated with autoimmune diseases, diabetes, sepsis, diffuse intravascular coagulation, tissue injury, hypotension, and death [22]. The inhibition of these inflammatory mediators employing pharmacological modulators has been used as an effective therapeutic strategy to reduce inflammatory reactions [23].

**Fig. 2:**
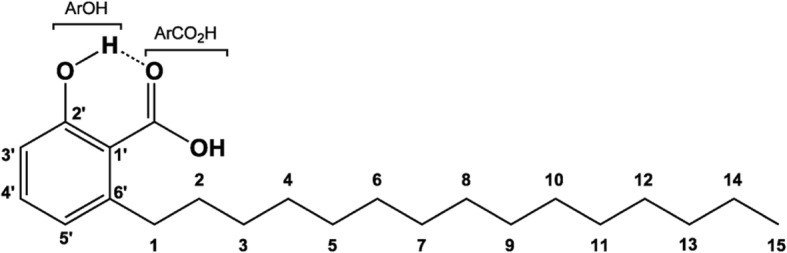
Chemical structure of LDT11 molecule

Considering that biosynthetic molecules derived from CNSL have been tested in cellular models in vitro [24, 25], the present work proposes to evaluate the anti-inflammatory profile of *Anacardium occidentale L.* phenolic lipid (LDT11, Fig. 2), in the cellular model. Results of this analysis may offer alternative therapeutic strategies for the treatment of inflammation.

## Methods

The production of inflammatory mediators was analyzed on RAW 264.7- TIB-71 murine macrophages cell culture (American Type Culture Collection - ATCC), previously treated with LDT11. Cells were purchased from the cell bank of the Adolf Lutz Institute (São Paulo, Brazil), and cultured according to the ATCC criteria.

### Synthesis and characterization of LDT11 as a potential anti-inflammatory agent

LDT11 is a derivative designed from cashew nut shell liquid (CNSL) phenolic lipid. Compounds from library of the Laboratory of Development of Therapeutic Innovations (LDT), part of the University of Brasília (Brazil) were used in this research. LDT11 synthesis was performed as follows: to a solution of the mixture of anacardic acids (5 g, 14.5 mmol for average molecular wt 344) in ethanol (50.0 mL) was added 10% palladium-carbon (0.2 g) and shaken in a Parr apparatus (Parr Instrument Company©, Moline, IL, USA), under hydrogen atmosphere (4 atm, 60 psi) at room temperature. After six hours, the mixture was filtered and the solvent was evaporated under reduced pressure. The residue was recrystallized from hexane to afford a saturated anacardic acid (LDT11) as a white solid (4,55 g, 90%, mp 81 °C–83 °C, Rf 0.48 – Hex:AcOEt 4:1). IR (KBr) ν_máx_ cm^− 1^: 3326 (ν_OH_); 2954 (ν_asCH3_); 2920 (ν_asCH2_); 2850 (ν_sCH2_); 1610 (ν_C=O_); 1560, 1542, 1498 e 1466 (ν_C=C_); 1287(ν_asC-O_); 1086 (ν_sC-O_); ^1^H NMR (300 MHz, CDCl_3_): δ 0.89 (t, *J* = 6.5 Hz, 3H, 15); 1.26 (m, 24H, 3–14); 1.60 (qi, *J* = 6.6 Hz, 2H, 2); 3.00 (t, *J* = 7.7 Hz, 2H, 1); 6.79 (d, *J* = 7.4 Hz, 1H, 3′); 6.89 (d, *J* = 8.0 Hz, 1H, 5′); 7.37 (t, *J* = 7.9 Hz, 1H, 4′); 10.72 (s, ArOH); ^13^C NMR (75 MHz, CDCl_3_): δ 14.3 (CH_3_, 15); 22.9 (CH_2_, 14); 29.6–30.0 (CH_2_, 3–12); 32.1 (CH_2_, 13); 32.2 (CH_2_, 2); 36.7 (CH_2_, 1); 110.6 (C, 1′); 116.1 (CH, 3′); 123.0 (CH, 5′); 135.6 (CH, 4′); 148.1 (C, 6′); 163.8 (C, 2′); 176.5 (ArCO_2_H).

The characterization of the molecule was carried out by the analysis of nuclear magnetic resonance (300 MHz Bruker Avance DRX NMR) of hydrogen and carbon-13, and by the infrared spectra (Spectrum BX, Perkin Elmer, Waltham, MA, USA). LDT11 was used for immunoprotective tests for inflammation in vitro*.* LDT11’s biological activity was compared to two commercial drugs: acetylsalicylic acid (ASA) (Sedalive, Vitamedic, Brazil), which has a similar chemical structure to LDT11, and corticosteroid dexamethasone (DEX) (Decadron, Aché Laboratórios Farmacêuticos S.A, Brazil).

### Quantitation of viable cell number - WST-8 assay

The cytotoxicity of the macrophages treated with synthetic phenolic derivatives was determined using the WST-8 assay (Cell Counting kit-8, Sigma-Aldrich, St. Louis, MO, USA). Cells were grown in complete Dulbecco’s Modified Eagle’s Medium (DMEM) at a concentration of 1 × 10^5^ in 96-well plates and incubated at 37 °C in an atmosphere of 5% CO_2_ for 48 h. The culture medium was subsequently exchanged for 100 μL of DMEM supplemented with 5% colorless fetal bovine serum (FBS), and 100 μL of LDT11 was added to the wells at concentrations of 25 μM, 50 μM, 75 μM, 100 μM, 125 μM, 150 μM, totaling a volume of 200 μL per well. After that, the plate was once more incubated under the previously described conditions.

The assay was performed using samples and controls in triplicate. After 48 h, the medium was discarded, and 100 μL of colorless DMEM, supplemented with 5% FBS was added, followed by the addition of 10 μL of WST-8 to each well. Non-stimulated cells were used as positive controls. Cell death control was performed using cells treated with 10 μL of 1% Triton-X. After an incubation of four hours with WST-8, the absorbance of the samples was measured in a TP-Reader microplate spectrophotometer (Thermoplate, Palm City, FL, USA) using a 450 nm wavelength filter.

### Neutral red uptake assay

The Neutral red uptake assay was performed following the protocol described by Tanner et al. [26], with some modifications. The assay was carried out under the same conditions as the WST-8 assay. After 48 h of incubation with the LDT11, the medium was discarded, the cells were washed twice with phosphate buffered saline solution (1X PBS, pH 7.4), and 100 μl of DMEM supplemented, and 50 μg/mL of neutral red was added to the wells. The plate was incubated under the conditions described in the item 2.2. The medium was then discarded, and the cells were washed five times with 1X PBS to remove the excess of dye; which was followed by the addition of 100 μl of alcohol-acid solution (50% ethanol, 1% acetic acid and 49% distilled water) to each well to fix the neutral red to the cells. The plate was shaken for 10 min, and the absorbance of the samples was read in a TP-Reader microplate spectrophotometer (Thermoplate, Palm City, FL, USA) with a 492 nm filter. The results were expressed as a percentage, the value obtained for the positive control (untreated cells), being considered as 100% viability. The equation used was: viability (%) = (number of viable cells / total number of untreated cells) × 100.

### Analysis of gene expression

To evaluate the influence of LDT11 ​​on RAW 264.7 gene expression, after stimulation with LPS, quantitative real-time polymerase chain reaction (qPCR) was performed. The RNA from cells was extracted, purified and quantified, and the resulting complementary DNA was prepared for the qPCR reaction, as follow: RAW 264.7 cells were cultured in six well plates at a concentration of 5 × 10^6^ cells/well, each containing 3 mL 10% complete DMEM medium. The plates were incubated (approximately 24 h) until an estimated confluence of 90% was obtained. The medium was discarded, and 3 mL of colorless DMEM medium without FBS supplementation was added in order restrain the cells growth rate. The immune-protective properties of LDT11 were analyzed in: (a) untreated cells (NT); (b) cells stimulated exclusively with LPS; (c) cells treated with LDT11, and subsequently exposed to LPS; (d) cells treated with ASA, and then exposed to LPS; (e) cells treated with DEX, and exposed to LPS.

### Extraction, purification, quantification of RNA and cDNA synthesis

For the extraction and purification of RNA, Direct-zol ™ RNA Miniprep Kit (R2051, Zymo Research, Orange, CA, USA), was used following the manufacturer’s recommendations. The quantification of extracted RNA was determined by using the NanoDrop 2000 spectrophotometer (Thermo Fisher Scientific, Waltham, MA, USA). The cDNA synthesis was performed using High-Capacity cDNA Reverse Transcription Kit (Applied Biosystems, Waltham, MA, USA), according to manufacturer’s instructions.

### Gene analysis and characterization

Genes involved in the inflammatory response were selected to test the biological activity of LDT11. Seven primers synthesized by Integrated DNA Technologies (Skokie, IL, USA) were used, as described in Table 1. The design of the primer pairs was performed using the Primer Express Program (Applied Biosystems, Waltham, MA, USA) based on sequences obtained from the Mouse Transcriptome Database (http://www.informatics.jax.org).

**Table 1:** RAW 264.7 macrophages genes analyzed in the study. Primers Description Sequences Mt.*

Primers	Description	Sequences	Mt*
*GAPDH*	Glyceraldehyde-3-phosphate dehydrogenase	5’-CCGGTGCTGAGTATGTCG-3′ 5’-CCCTGTTGCTGTAGCCGTA-3’	85,72
*COX-2*	Cyclooxygenase 2	5’-TGAGTACCGCAAACGCTTCTC-3′ 5’-TGGACGAGGTTTTTCCACCAG-3’	80,35
*IL-1β*	Interleukin 1 beta	5’-TGAAATGCCACCTTTTGACAG-3′ 5’-CCACAGCCACAATGAGTGATA-3’	54,80
*IL-6*	Interleukin 6	5’-GCTACCAAACTGGATATAATCAGGA-3′ 5’-CAGGTAGCTATGGTACTCCAGAA-3’	73,48
*iNOS*	Induced nitric oxide synthase	5’-GGCAGCCTGTGAGACCTTTG-3′ 5’-GCATTGGAAGTGAAGCGTTTC-3’	77,81
*NF-κB*	Nuclear Factor kappa b	5’-AGCCAGCTTCCGTGTTTGTT-3′ 5’-AGGGTTTCGGTTCACTAGTTTCC-3’	77,81
*TNF-α*	Tumor necrosis factor alpha	5’-TCTTCTCATTCCTGCTTGTGG-3′ 5’-GGTCTGGGCCATAGAACTGA-3’	81,70

*Mt Melting temperature (°C)

### Quantitative real-time polymerase chain reaction

The Quantitative Real-Time Polymerase Chain Reaction (qPCR) was performed in triplicates, using SYBR Green system (Absolute qPCR SYBR Green Rox Mix - Thermo Fisher Scientific Inc., Vilnius, Lithuania, USA) in a solution containing 50 ng of cDNA, 5 pmol of each primer forward and reverse and QSP of ultrapure water, totalizing 20 μL. Amplification assays were performed on a StepOnePlus Real-Time PCR System (Applied Biosystems, Carlsbad, CA, USA) under the following conditions: initial denaturation temperature at 95 °C for 10 min, 40 cycles of denaturation at 95 °C for 15 s, annealing and extension at 60 °C for 1 min and 72 °C for 30 s. All reactions were performed three times for each gene, and *GAPDH* gene was used both as endogenous control and normalizing gene. Water was used as negative control substituting cDNA.

The result, expressed in CT value, refers to the number of cycles in qPCR required for the fluorescent signal to reach the threshold detection. The analysis of the gene expression of the inflammatory markers was obtained by the relative quantification of their transcripts by the Delta-Delta Ct (ΔΔCt) method, which allows a relative comparison with the group that did not receive treatment (NT) and cells stimulated with LPS. The melting curve was used as quality control of amplification products.

For the data analysis, the values ​​found for the control group, LPS- stimulated cells, were considered 100% inflamed. The other tests, with their respective treatments (LDT11, ASA and DEX) were analyzed comparing with the inflamed group.

### Nitric oxide quantification

For the analysis of Nitric Oxide **(**NO), was used the Griess method, by adding 100 μL of Griess reagent (1% [*w*/*v*] sulfanilamide, in a 5% phosphoric acid, and 0.1% [w / v] N-1- naphthyl-ethylenediamide dihydrochloride (NEED) in water). Samples of culture supernatants were analyzed by microplate reader (TP-Reader microplate spectrophotometer, Thermoplate, Palm City, FL, USA), using the spectrum 450 nm, and the results expressed in μmol/L of NO - comparing the optical density (OD) obtained with a standard curve of NO - ranging from 1.56 μM to 100.0 μM.

### IL-6 quantification

The quantification of Interleukin-6 (IL-6) was performed by applying the competitive immunoenzymatic assay, as established in the manufacturer’s kit (#27768 Mouse IL-6 Kit – Immuno-Biological Laboratories Co., Ltda, Hamburg, Germany). The same treatment used in the gene expression assessment was applied in this assay, using the supernatants of RAW 264.7 cells treated with the synthetic phenolic derivative LDT11 at 50 μM concentration.

### Statistical analysis

Statistical analysis was performed using the Analysis of Variance (ANOVA) test, followed by Post-Hoc tests (Bonferoni, Dunnett and t-test) when applicable, according to GraphPad Prism version 4.0 (GraphPad Software, San Diego, USA). Statistically significant differences were considered when *p* < 0.05.

## Results

### Cytotoxicity analysis and quantitation of viable cell number

The determination of the cytotoxic effect of LDT11 by the WST-8 and Neutral Red methods was performed using the following concentrations: 25 μM, 50 μM, 75 μM, 100 μM, 125 μM, and 150 μM.

### WST-8 assay

**Fig. 3:**
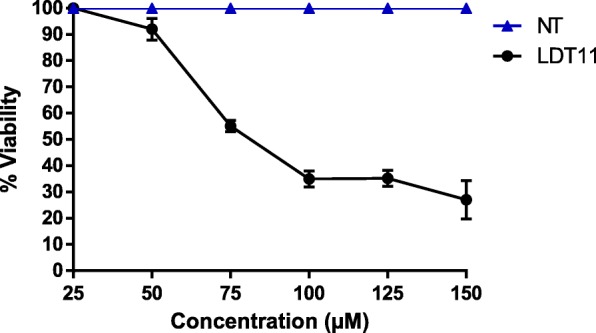
Cell viability by the WST-8 method in RAW264.7 cell line, using different concentrations of the phenolic acid derivative LDT11. NT - untreated cells. LDT11 - cells treated with LDT11 and then with LPS

LDT11 showed approximately 100% cell viability in a concentration of 25 μM. The cell viability declined to 90% with a LDT11 concentration of 50 μM. Concentrations equal or over 75 μM ensued a cell viability equal or lower than 60% and were considered cytotoxic (Fig. 3).

### Neutral red uptake assay

**Fig. 4:**
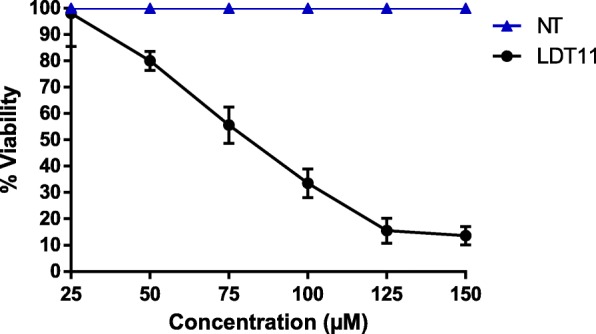
Cell viability test by the Neutral Red Uptake Assay in RAW264.7 cell line, using different concentrations of the phenolic derivative, LDT11. NT - untreated cells. LDT11 - cells treated with LDT11 and then with LPS

Neutral Red Uptake Assay was performed to confirm the cell viability obtained by the WST-8 assay on the RAW264.7 cell line, using similar LDT11 concentrations. The assay showed results similar to those found by the WST-8 method (Fig. 4).

From the results of the cell viability assays, toxic concentrations of LDT11 were excluded from the study. The standardized concentration for the other tests was 50 μM. For further comparison, the following assays with ASA and DEX were also made using the same concentration established for LDT11, 50 μM.

### Modulation and relative quantification of transcripts of inflammatory genes

Gene modulation of the inflammatory process was analyzed after interaction with the CNSL derivative, LDT11. Gene expression was evaluated by the relative quantification of *TNF-α, COX-2, iNOS, NF-kB, IL-1β* and *IL-6* genes in RAW264.7 cells. Inflammation was induced with 1 μg/mL LPS, both in control cells and in LDT11 treated cells. The analysis of the results was carried out 6 and 24 h post- treatment.

**Fig. 5:**
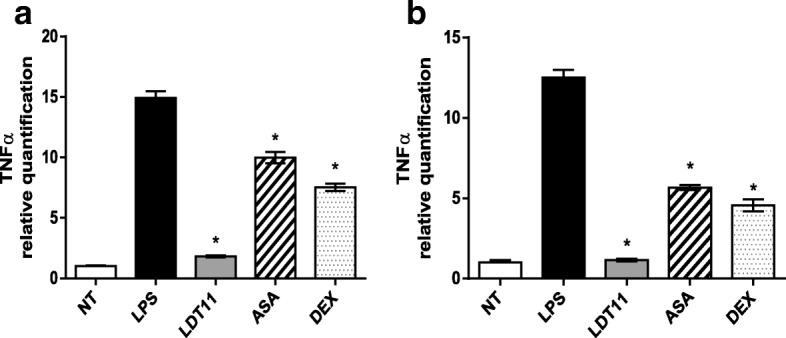
Determination of the relative amount of *TNF-α* gene transcripts in RAW 264.7 cell line for the evaluation of inflammatory response at 6 (**a**) and 24 h (**b**) post-stimulus. NT- untreated cells; LPS - LPS-stimulated cells; LDT11 - cells treated with LDT11 and then with LPS; AAS - cells treated with commercial acetylsalicylic acid and then with LPS; DEX - cells treated with the commercial anti-inflammatory Dexamethasone and then with LPS. All data are from at least three independent experiments, *p < 0.05, statistically different compared with cells LPS-stimulated

As seen in Fig. 5, the gene expression of *TNF-α*, six hours after interaction with LDT11, showed an eight-fold decrease (88%) in comparison with the gene expression disclosed by the control cells (LPS). The results obtained from the interaction with ASA and dexamethasone (DEX) were respectively one and a half times lower (33%) for ASA and two times lower (50%) for DEX (Fig. 5a). The results obtained 24 h after treatment were similar with those obtained after six hours. Both had a decrease in gene expression in all treatments analyzed. Cells treated with LDT11 disclosed 10 times (91%) less gene expression than the positive control, while cells treated with ASA and DEX respectively showed a gene expression two and three times lower (55 and 64%) than control cells, Fig. 5b.

**Fig. 6:**
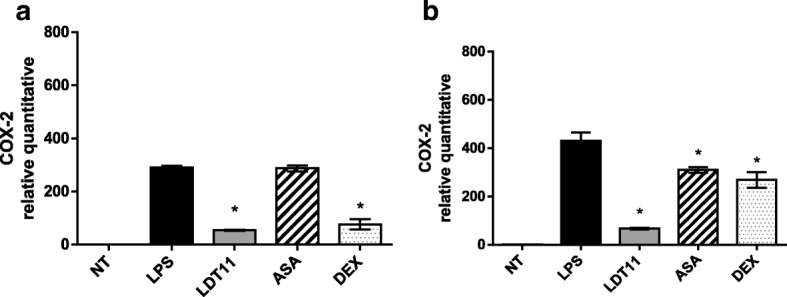
Determination of the relative amount of *COX-2* gene transcripts in RAW 264.7 cell line for the evaluation of inflammatory response at 6 (**a**) and 24 h (**b**) post-stimulus. NT- untreated cells; LPS - LPS-stimulated cells; LDT11 - cells treated with LDT11 and then with LPS; AAS - cells treated with commercial acetylsalicylic acid and then with LPS; DEX - cells treated with the commercial anti-inflammatory Dexamethasone and then with LPS. All data are from at least three independent experiments, *p < 0.05, statistically different compared with cells LPS-stimulated

*COX-2* was the second gene analyzed (Fig. 6). After six hours of treatment with LDT11, *COX-2* gene expression decreased more than five-fold (81%) when compared to control cells (Fig. 6a). Cells treated with ASA did not show a significant difference, and those treated with DEX showed a four-fold decrease (74%) in gene expression when compared to control cells. After 24 h of interaction with LTD11 (Fig. 6b), the gene expression disclosed a six-fold decrease (84%) in treated cells than in control cells. The reduction in gene expression caused by both ASA and DEX was approximately 2-fold higher (30%) than that observed in control cells.

**Fig. 7:**
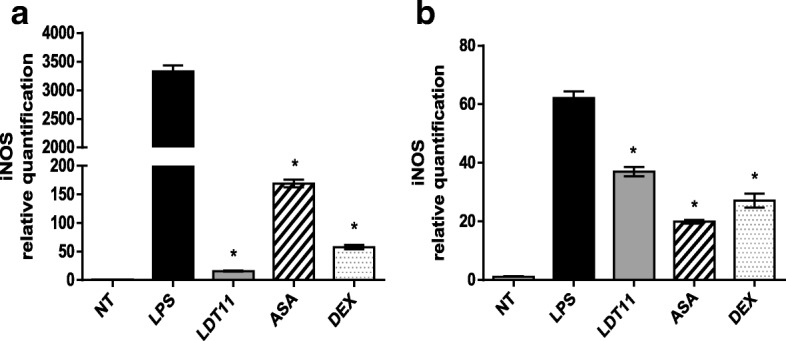
Determination of the relative amount of *iNOS* gene transcripts in RAW 264.7 cell line for the evaluation of inflammatory response at 6 (**a**) and 24 h (**b**) post-stimulus. NT- untreated cells; LPS - LPS-stimulated cells; LDT11 - cells treated with LDT11 and then with LPS; AAS - cells treated with commercial acetylsalicylic acid and then with LPS; DEX - cells treated with the commercial anti-inflammatory Dexamethasone and then with LPS. All data are from at least three independent experiments, *p < 0.05, statistically different compared with cells LPS-stimulated

After six hours of LTD11 treatment, the expression of the *iNOS* gene, (Fig. 7) showed a 200 times decrease (100%) comparing with control cells. Treatment with ASA resulted 20-fold (99%) decreased expression, while cells treated with DEX showed a 60-fold (98%) gene suppression (Fig. 7a). After 24 h (Fig. 7b), both LDT11 and DEX treated cells showed approximately a 2-fold decreased gene expression (50%) while a threefold decrease (68%) was observed in ASA-treated cells.

**Fig. 8:**
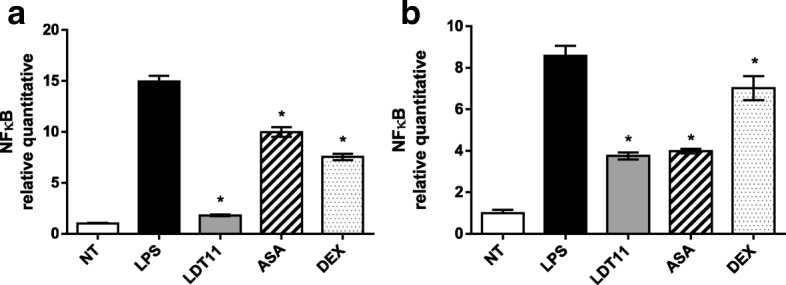
Determination of the relative amount of *NF-kB* gene transcripts in RAW 264.7 cell line for the evaluation of inflammatory response at 6 (**a**) and 24 h (**b**) post-stimulus. NT- untreated cells; LPS - LPS-stimulated cells; LDT11 - cells treated with LDT11 and then with LPS; AAS - cells treated with commercial acetylsalicylic acid and then with LPS; DEX - cells treated with the commercial anti-inflammatory Dexamethasone and then with LPS. All data are from at least three independent experiments, *p < 0.05, statistically different compared with cells LPS-stimulated

The action of LTD11 on the *NF-kB* gene expression can be seen in Fig. 8. In comparison with control group, cells treated with LTD11 resulted an eight-fold (88%) decrease in *NF-kB* gene expression. The treatment of the cells with ASA and DEX produced respectively a decrease in gene expression equivalent to 1.5-fold (33%) for the first and two-fold (50%) for the second drug (Fig. 8a). In the treatment performed after 24 h (Fig. 8b), both LDT11 and ASA treated cells showed a decrease of about two-fold (55%) in the *NF-kB* gene expression. On the other hand, cells treated with DEX failed to show any significant difference when compared to the control cells.

**Fig. 9:**
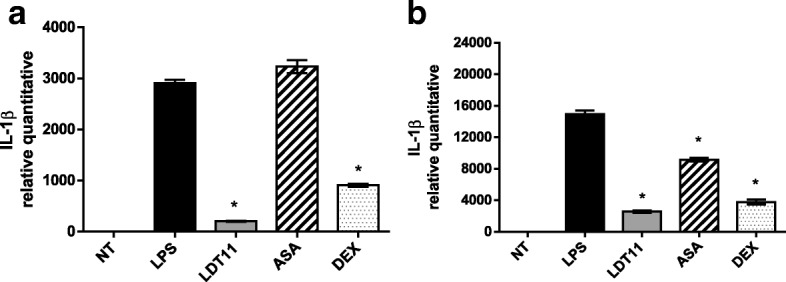
Determination of the relative amount of *IL-1β* gene transcripts in RAW 264.7 cell line for the evaluation of inflammatory response at 6 (**a**) and 24 h (**b**) post-stimulus. NT- untreated cells; LPS - LPS-stimulated cells; LDT11 - cells treated with LDT11 and then with LPS; AAS - cells treated with commercial acetylsalicylic acid and then with LPS; DEX - cells treated with the commercial anti-inflammatory Dexamethasone and then with LPS. All data are from at least three independente experiments, *p < 0.05, statistically different compared with cells LPS-stimulated

The *IL-1β* gene also showed decreased expression in most of the assays (Fig. 9). After a six-hour interaction with LDT11, *IL-1β* gene expression decreased more than 14-fold (93%). Treatment with ASA did not cause a significant decrease in its expression whereas DEX led to a three-fold reduction (69%) when compared to control cells (Fig. 9a). After 24 h (Fig. 9b), when compared with control cells, those treated with LDT11 showed an approximately six-fold decrease (83%) in gene expression, whereas ASA and DEX caused respectively a two-fold (39%) and five-fold (75%) decline.

**Fig. 10:**
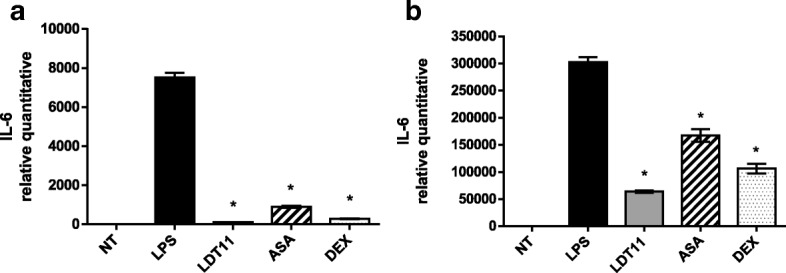
Determination of the relative amount of *IL-6* gene transcripts in RAW 264.7 cell line for the evaluation of inflammatory response at 6 (**a**) and 24 h (**b**) post-stimulus. NT- untreated cells; LPS - LPS-stimulated cells; LDT11 - cells treated with LDT11 and then with LPS; AAS - cells treated with commercial acetylsalicylic acid and then with LPS; DEX - cells treated with the commercial anti-inflammatory Dexamethasone and then with LPS. All data are from at least three independente experiments, *p < 0.05, statistically different compared with cells LPS-stimulated

After six hours of treatment with LDT11 there was a 65-fold (98%) decrease in the gene expression of *IL-6*, in comparison with the control cells. Gene expression of *IL-6* decreased respectively 8-fold (88%) in ASA-treated cells and 27-fold (96%) in DEX-treated cells (Fig. 10a). After 24 h (Fig. 10b) the gene expression decreased 5-fold (79%) in the cells treated with LDT11 while the decrease was respectively two-fold (45%) in the cells treated with ASA and three-fold (65%) in the cells treated with DEX.

### Assessment of nitric oxide and cytosine IL-6

**Fig. 11:**
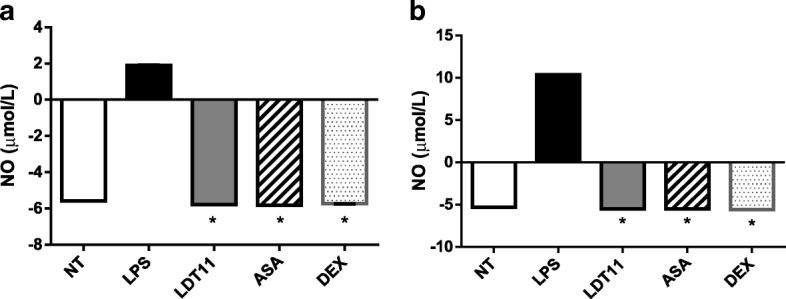
Evaluation of the potential immunoprotective effect of the CNSL derivatives by measuring the levels of nitric oxide production in the supernatant at 6 h (**a**) and 24 h (**b**) post-stimulus. NT- untreated cells; LPS - LPS-stimulated cells; LDT11 - cells treated with LDT11 and then with LPS; AAS - cells treated with commercial acetylsalicylic acid and then with LPS; DEX - cells treated with the commercial anti-inflammatory Dexamethasone and then with LPS. All data are from at least three independente experiments, *p < 0.05, statistically different compared with cells LPS-stimulated

Nitric oxide, resulting from the presence of oxygen and nitrogen reactive metabolites produced during the inflammatory process, causes an increase in the oxidative stress of the cells. The presence of NO in the supernatant of RAW 264.7 cells was measured to confirm the influence of LDT11 on anti-inflammatory activity. The following assays were performed with the same timing (six and 24 h) of the previous analysis. The LDT11 was able to protect RAW264.7 cells against oxidative stress by reducing NO production by about eight times (95%) after six hours, and about 15 times (100%) after 24 h (Fig. 11).

**Fig. 12:**
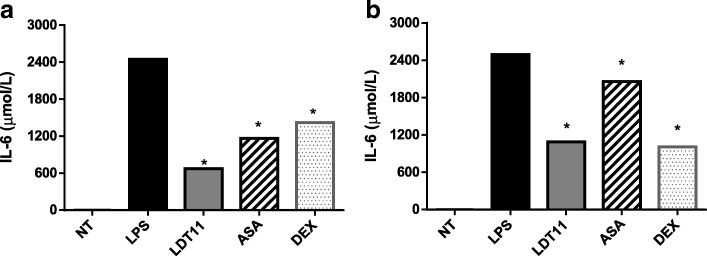
Evaluation of the potential immunoprotective effect of the CNSL derivatives by measuring the levels of cytosine *IL-6* production in the supernatant at 6 h (**a**) and 24 h (**b**) post-stimulus. NT- untreated cells; LPS - LPS-stimulated cells; LDT11 - cells treated with LDT11 and then with LPS; AAS - cells treated with commercial acetylsalicylic acid and then with LPS; DEX - cells treated with the commercial anti-inflammatory Dexamethasone and then with LPS. All data are from at least three independent experiments, *p < 0.05, statistically different compared with cells LPS-stimulated

Additionally, to confirm the results obtained in the analysis of gene expression, the cytosine *IL-6* was also assayed. The pre-treatment of the cells with LTD11 resulted in a significant protective effect against inflammation, reducing *IL-6* production by more than 1700-fold (76%) after six hours (Fig. 12a) and more than 1400-fold (60%) after 24 h (Fig. 12b). Cells treated with ASA reduced *IL-6* production respectively by more than 120-fold (52%), after six hours and by more than 400 (17%) after 24 h. Treatment with DEX reduced the *IL-6* production at about 1000-fold (42%) after six hours and at about 1400-fold (60%) after 24 h.

## Discussion

The present study evaluated the immunoprotective effect of LDT11; a compound synthesized from the anacardic acid extracted from the CNSL. This evaluation was performed through the quantification of gene transcripts involved in the inflammatory response, and the measurement of NO and IL-6 production in RAW 264.7 murine macrophages. The murine macrophages culture was firstly treated with LDT11 and posteriorly stimulated with LPS that mimicked the inflammatory response and induced high production cytosine and oxidative stress [27–29]. Overproduction of pro-inflammatory mediators such as TNF-α, COX-2, iNOS, NF-κB, IL-1β and NO has been implicated in several inflammatory diseases [27, 29, 30]. Therefore, an agent that prevents the release of these mediators or downregulates the expression of these cytokines may be an valuable therapeutic strategy for preventing inflammatory reaction [27, 28], turning LDT11 a potential candidate for the formulation of new drugs.

In this study, two different colorimetric methods, the WST-8 assay, and the neutral red uptake test, were performed to evaluate if the LDT11 used in the experiments would show some degree of cytotoxicity. The analysis of its in vitro cytotoxicity allowed the determination of the concentrations needed to obtain the best performance of its biological activity without compromising the cellular viability. Viability and cytotoxicity results were similar for both tests at all concentrations used (25 μM, 50 μM, 75 μM, 100 μM, 125 μM, and 150 μM). At concentrations of 25 μM and 50 μM, cell viability was greater than 80%. However, the increase of the concentrations to 75 μM, 100 μM, 125 μM and 150 μM caused more than 60% decrease in cell viability, being consequently considered cytotoxic. Others studies found that the assay shows cytotoxic activity when cell death exceeds 50% [18, 31]. In the present research values ​​above 60% were considered citotoxic. Therefore, the concentration chosen to continue the experiments of gene expression analysis was 50 μM of LDT11.

The *TNF-α* gene has the main physiological effect of promoting the inflammatory immune response through the recruitment and activation of neutrophils and monocytes. Consequently, TNF-α is responsible for a several effects on the body, promoting vasodilation and acting on endothelial cells, stimulating the secretion of a group of cytokines that have chemotactic action on leukocytes. TNF-α is also the cytokine responsible for septic shock, in addition to inhibiting the appetite and inducing fever through the release of the adrenocorticotrophic hormone (ACTH) [30, 32]. Additionally, it is also a major inducer of the transcription factor NF-κB, and degrades the inhibitor of NF-κB (IκB). Degradation of IκB allows NF-κB to be translocated to the nucleus. [21, 33]. Pretreatment with LDT11 showed suppression of the *TNF-α* gene in LPS-stimulated cells greater than the suppression observed in cells treated with commercial anti-inflammatory drugs. Consequently, a similar suppression was observed in *NF-κB* and *TNF-α*, being greater in LDT11-treated cells than in the cells treated with commercial drugs.

NF-κB is responsible for the transcription of innumerable genes related to pro-inflammatory activity, such as *IL-1, IL-6, iNOS, COX-2* [34–36]. Consequently, a decrease in the gene expression of these inflammatory mediators was also observed. Compared with LPS-stimulated cells, the *COX-2* and *IL-1β* genes showed more than 80% gene suppression after treatment with LDT11. The suppression of *iNOS* and *IL-6* genes was practically total in LDT11 pre-treated cells.

The evaluation of the treatment after 24 h showed that the relative amount of *iNOS* decreased in all the tests performed, including in the LPS treated cells. Consequently, the gene suppression was less expressive than that observed in experiments conducted after six hours. Gene expression of *iNOS* after 24 h was the only outcome in which treatment with LDT11 was inferior to the results obtained with the commercial drugs. Although, treatment with LDT11 was also capable of reducing the *iNOS* gene expression when compared to LPS treated cells.

Nitric oxide (NO), an important molecule produced in the process of oxidative stress, is synthesized by the enzyme nitric oxide synthetase, that is a product of the *iNOS* gene. It acts as a biological mediator similar to neurotransmitters and can regulate the tonus of blood vessels. On the other hand, it is an oxygen free radical that can function as a cytotoxic agent in pathological processes, especially in inflammatory diseases [37–39].

Interleukin-6 (IL-6) is one of the leading mediators of the acute phase of inflammation, with a crucial activity on eosinophil chemotaxis to the inflammation site, and has a vital role on coagulation [20, 40]. IL-6 is known as a multifunctional cytokine, which in addition to its pro-inflammatory and sclerosing functions, also affects the activity of neoplastic cells [30, 41]. In addition to its critical local effects, this cytokine has systemic activity, which contributes to the defense of the organism. One of these effects is the elevation of body temperature, causing fever from an endogenous source [21].

Therefore, NO and IL-6 were measured to confirm the results found with the analysis of the transcripts obtained by qPCR. The assays were performed using the supernatants of cell cultures submitted to the treatment already described on 2.4 item. The potential immune-protective activity of LDT11 was evidenced by the results obtained, both at 6 LPS treated and at 24 h post-treatment, which demonstrated a significant reduction in the production of NO and IL-6 when compared to LPS treated cells.

The evaluation of expression of the genes *TNF-α, COX-2, iNOS, NF-κB, IL-1β* and *IL-6* at 6 and 24 h after treatment of the cells with LDT11, makes evident that the LDT11 was most effective in protecting against the inflammation when compared with results obtained with the use of commercial drugs.

In addition to a significant reduction in the expression of inflammatory genes, LDT11 also had a faster and superior anti-inflammatory action than the commercial products, and its response was already evident in the test carried out six hours after the treatment of the cells, suggesting that this molecule can be used as an anti-inflammatory drug.

## Conclusion

LDT11, a phenolic derivative of CNSL, showed potential immunoprotective and anti-inflammatory properties, having a rapid and effective activity. Treatment with LDT11 decreased the expression of the *TNF-α, COX-2, iNOS, NF-κB, IL-1β* and *IL-6* inflammatory genes. Additionally, LDT11 protective effect on inflammation was confirmed by the decreased of NO and IL-6 production. The anti-inflammatory activity of LDT11 was superior to commercial drugs, ASA and DEX.

## Abbreviations

ANOVA: Analysis of variance
ASA: acid acetylsalicilic
ATCC: American Type Culture Collection
cDNA: complementary DNA sequence
CNSL: cashew nut shell liquid
CO_2_: Carbon dioxide
COX-2: cyclooxygenase isoform 2
CT: Crossing threshold
DEX: Dexamethasone
DMEM: Dulbecco’s Modified Eagle Medium
DMSO: Dimethyl Suffoxide
DNA: Deoxyribonucleic acid
DNAse: Deoxyribonuclease
dNTP: Deoxynucleotides 5′-triphosphate
ELISA: Enzyme Linked Immuno Sorbent Assay
FC: Fold-change
GAPDH: Glyceraldehyde-3-phosphate dehydrogenase
IL-1β: Interleukin-1β
IL-6: Interleukin-6
iNOS: Nitric oxide synthase induced
LDT11: semisynthetic molecule 11 - of the Laboratory of Development of Therapeutic Strategies
LDT13: semisynthetic molecule 13 - of the Laboratory of Development of Therapeutic Strategies
LPS: Lipopolysaccharide
NEED: N- (1-Naphthyl) ethyl-enedinamine
NF-κB: Nuclear transcription factor κB
NO: Nitric oxide
OD.: Optical Density
p: statistical significance
PBS: Phosphate buffered saline
pH: Hydrogen ionic potential
PMN: Polymorphonuclear
RNA: Ribonucleic acid
RNAse: Ribonuclease
RT: Reverse Transcription
SBF: Fetal bovine sérum
SYBR Green or SyBrgreen: Fluorescent dye that attaches to DNA tape
Tm: Melting temperature or dissociation temperature
TNF-α: Tumor Necrosis Factor alpha (α)
WST-8: 5-(2,4-disulfophenyl)-3-(2-methoxy-4-nitrophenyl)-2-(4-nitrophenyl)-2H-tetrazolium, inner salt, monosodium salt
